# B Cells Participate in Thymic Negative Selection of Murine Auto-reactive CD4^+^ T Cells

**DOI:** 10.1371/journal.pone.0015372

**Published:** 2010-10-20

**Authors:** Friederike Frommer, Ari Waisman

**Affiliations:** Institute for Molecular Medicine, University Medical Center of the Johannes Gutenberg-University Mainz, 55131 Mainz, Germany; New York University, United States

## Abstract

It is well documented that thymic epithelial cells participate in the process of negative selection in the thymus. In recent years it was reported that also dendritic cells enter the thymus and contribute to this process, thus allowing for the depletion of thymocytes that are specific to peripherally expressed self-antigens. Here we report that also B cells may take part in the elimination of auto-reactive thymocytes. Using a unique mouse model we show that B cells induce negative selection of self-reactive thymocytes in a process that leads to the deletion of these cells whereas regulatory T cells are spared. These findings have direct implication in autoimmunity, as expression of a myelin antigen by B cells in the thymus renders the mice resistant to autoimmune inflammation of the CNS.

## Introduction

A critical step in the development of immunological tolerance is the exposure of immature T cells to self-peptide/MHC in the thymus. The clonal deletion of auto-reactive thymocytes in this process (negative selection) is one way to achieve tolerance and is supposed to be the major mechanism of immunological tolerance, given the fact that mice and humans deficient for the transcriptional regulator AIRE show an impaired clonal deletion in the thymus and suffer from multiorgan autoimmune disease [1], [2], [3], [4]. Studies in different mouse models [5], [6] have shown that particularly medullary thymic epithelial cells (mTECs) present a plethora of different autoantigens that are otherwise expressed in a tissue-specific manner. This phenomenon referred to as promiscuous gene expression is crucial for thymic negative selection of self-reactive T cells [7], [8]. Besides mTECs, BM-derived MHCII-expressing antigen presenting cells (APC) such as macrophages, dendritic cells (DCs) and also B cells reside the thymus and are thus potential participants in negative selection. Thymic macrophages were shown to be most likely unable to perform negative selection of autoreactive thymocytes [9] but they were described to rather function in the clearing of apoptotic cells [10], [11]. In addition to mTECs, also DCs were proposed to be important contributors to negative selection in the thymus [12], [13]. To evaluate the role of DCs in negative selection, MHCII^−/−^BM chimeras were used [14], [15]. Mice with an absent antigen presentation by DCs due to the lack of MHCII show an impaired negative selection of CD4^+^ thymocytes, indicated by the fact that MHCII^−/−^ chimeras exhibit increased numbers of self-reactive CD4^+^ thymocytes [16]. Another study using mice depleted of DCs shows an impaired negative selection of CD4^+^ T cells [17]. Additionally, DCs were also demonstrated to be able to participate in negative selection of CD8^+^ T cells [18].

The role of B cells in negative selection of thymocytes was only scarcely studied before. These cells were shown to participate in partial negative selection of CD4^+^ T cells [18]. In this study, a B cell-specific expression system of the MHCII I-Eα gene in an I-E negative background was used and thereby shown that thymic B cells are able to negatively select self-I-E-reactive CD4^+^ T cells [18].

To further address the importance of B cells in negative selection of autoreactive thymocytes and the generation of regulatory T cells in the thymus we made use of a mouse model that is characterized by the presentation of the self-peptide 35–55 of myelin oligodendrocyte glycoprotein (MOGp35-55) specifically on MHCII of B cells (B^MOG^ mice; [19]). These mice were crossed to MOG-specific T cell receptor (TCR) tg mice (2D2 mice; [20]) resulting in a mouse strain that combines the specific presentation of MOGp35-55 on B cells with a MOG-specific T cell repertoire. The presentation of MOGp35-55 on all B cells including thymic B cells in this mouse model makes it suitable to study the impact of B cells on thymocytes and their role in thymic negative selection. In these mice we observed a strong deletion of MOG-specific CD4^+^ transgenic T cells leading to the nearly entire absence of transgenic T cells in the periphery. In contrast, regulatory T cells seemed to be exempted from deletion as the proportion of CD4^+^Foxp3^+^ T cells in these mice was found to be increased.

## Results

### B cell-specific presentation of MOG peptide in the thymus leads to negative selection of MOG-specific T cells

**Figure 1:**
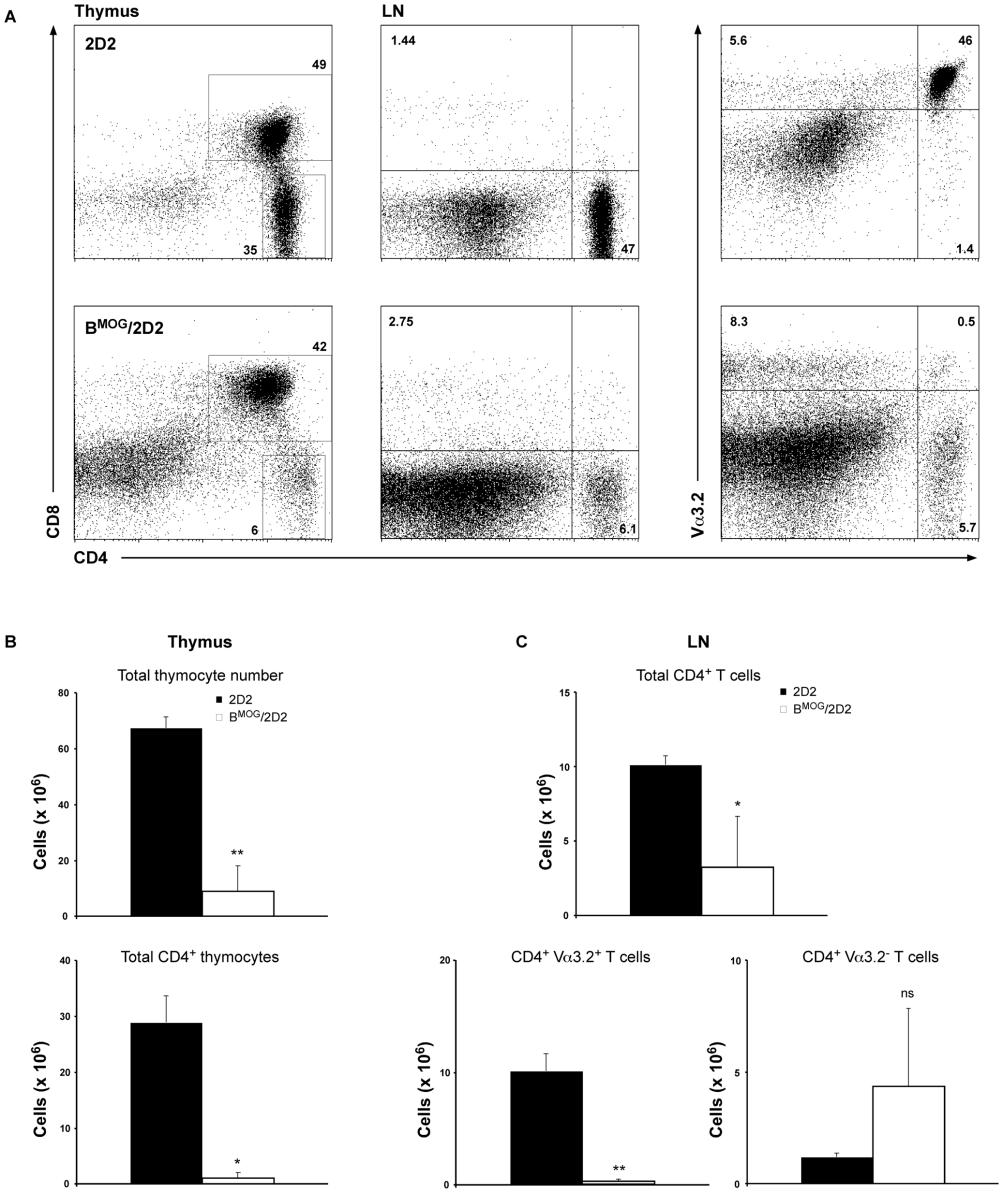
Deletion of 2D2 CD4^+^ T cells upon MOG encounter by B cells in the thymus. Thymus and LN from WT 2D2 (upper row) and B^MOG^/2D2 (lower row) mice were analyzed by FACS analysis for presence of CD8^+^ and CD4^+^ Va3.2^+^ T cells, respectively (A). Genotypes and antibodies used are as indicated. Cell surface markers are shown as coordinates. Cells were gated on live lymphocytes. Numbers besides gates or in quadrants indicate percent positive cells in each. Total and CD4 single positive thymocyte numbers (B) and LN total CD4^+^ as well as CD4^+^ Va3.2^+^ and CD4^+^ Va3.2^−^ T cell numbers (C) of 2D2 and B^MOG^/2D2 mice were calculated. Values represent mean ± SEM. ns, not significant.

Of all cells that express constitutively MHC class II molecules (MHCII), B cells are the most abundant in the body. In the mouse, B cells outnumber the other professional antigen presenting cells (APCs) in almost all tissues. B cells can also be found in the thymus, where most of them acquire a phenotype resembling B-1a cells, namely the expression of CD5 and CD44 [21]. We have previously demonstrated that B cells, when forced to present an autoantigen, can participate in the process of peripheral tolerance [19]. Once B cells encounter naïve T cells that recognize the peptide presented on MHCII, these T cells will become only partially activated and subsequently sensitive to the process of antigen-induced cell death. As B cells are also present in the thymus, we set to investigate whether they may participate also in the process of central tolerance. To answer this question, we made use of the B^MOG^ mice, where B cells constitutively present the MOG peptide p35-55 on MHCII [19]. These mice were crossed to 2D2 mice [20] that express a MOGp35-55-specific T cell receptor (TCR), forming the B^MOG^/2D2 mouse strain. To investigate whether constitutive expression of an autoantigen influences the process of thymocyte education, we analyzed thymi of B^MOG^/2D2 mice compared to thymi of 2D2 single transgenic mice. As can be seen in Fig. 1A, about half of the cells in the thymus of 2D2 mice are double positive (DP) for CD4 and CD8, and a third of the cells are CD4 single positive (SP). The relatively low percentage of DP and high percentage of CD4 SP T cells reflects, most likely, the very high efficiency of the MOG-specific TCR expression in these mice, as the latter was originally isolated from a CD4^+^ T cell clone [20]. In contrast, the number of SP CD4 T cells in the thymi of B^MOG^/2D2 mice was drastically reduced (Fig. 1A and B). Importantly, we also found a drastic reduction of CD4^+^ T cells in the lymph nodes (LN) (Fig. 1A and C) as well as spleens (not shown) of B^MOG^/2D2 mice. The total cell number of CD8^+^ T cells in these mice was only slightly but not significantly increased (data not shown). Further, we observed that the number of CD4 T cells that do not express the transgenic Vα3.2 chain is slightly elevated in LN of B^MOG^/2D2 mice when compared to 2D2 single transgenic mice (Fig. 1C). These findings suggest that B cells presenting self-antigen participate in the process of negative selection in the thymus by inducing the death of self-reactive T cells. In addition, these data indicate that during this process no significant levels of TCR editing can be found, as the latter should lead to a significant expansion of CD8^+^ and CD4^+^Vα3.2^−^ T cells in both thymus and LN.

### Negative selection of 2D2 T cells is due to MOG presentation by B cells and not by unspecific Cre activity of other thymic APCs

**Figure 2 pone-0015372-g002:**
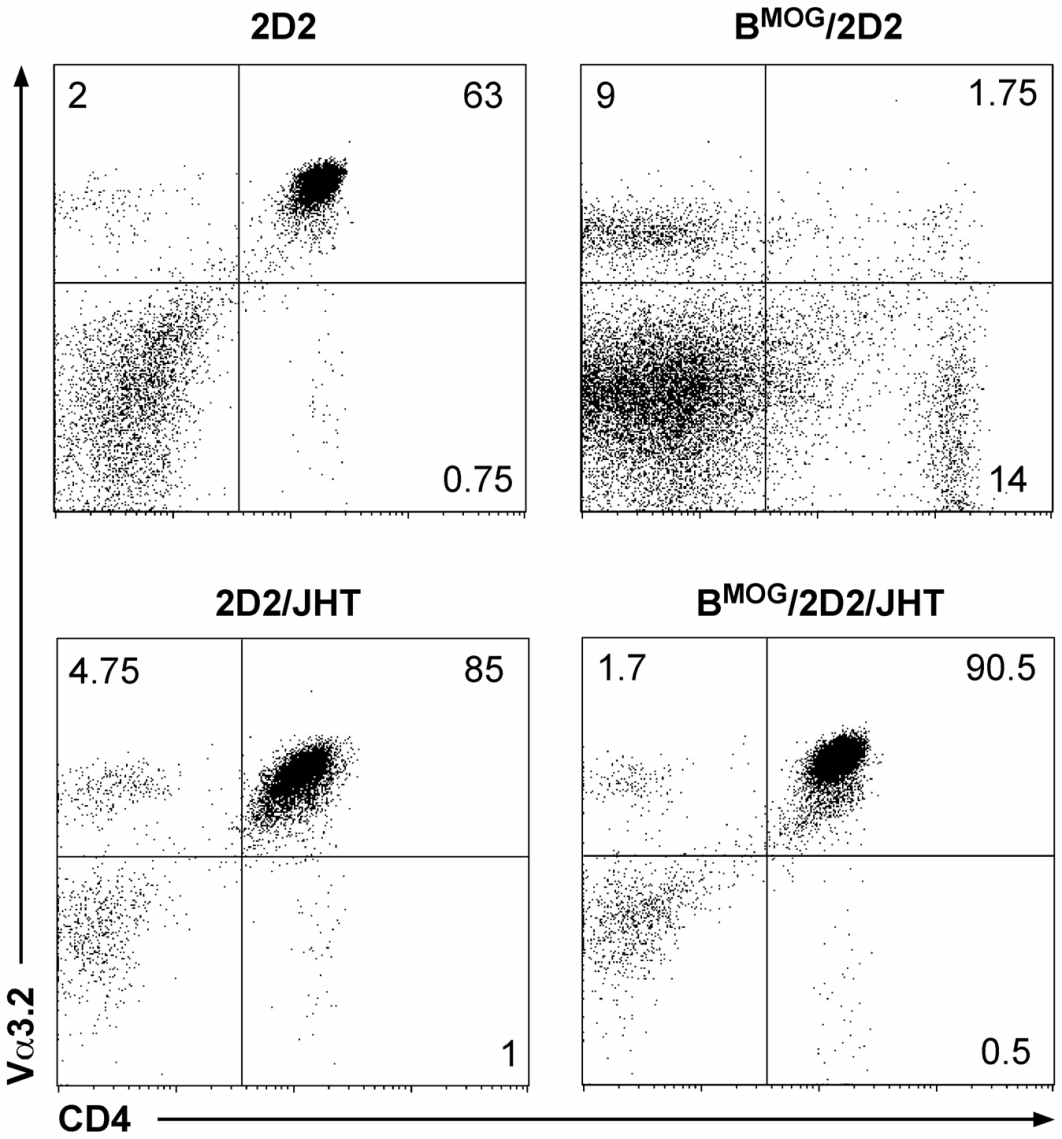
Deletion of 2D2 CD4^+^ T cells in the thymus is specifically induced by B cells. LN of 2D2 and 2D2/JHT (left row) as well as B^MOG^/2D2 and B^MOG^/2D2/JHT (right row) mice were analyzed by FACS analysis for presence of CD4^+^ Vα3.2^+^ T cells. Genotypes and antibodies used are as indicated. Cell surface markers are shown as coordinates. Cells were gated on live lymphocytes. Numbers besides gates or in quadrants indicate percent positive cells in each.

We were wondering whether the very efficient negative selection we observed in the thymi of B^MOG^/2D2 mice is in fact the result of peptide expression by other APCs in the thymus. Previously, we have ruled out the possibility that DCs and macrophages in the B^MOG^ mice present the MOG peptide, as we found that they were not able to trigger 2D2 TCR expressing cells *in vitro* [19]. But in the thymus, there are specialized cells, such as medullary thymic epithelial cells (mTEC) that participate in the process of negative selection, implicating the risk of unspecific MOG presentation in the thymus. To investigate whether the Cre-recombinase in B^MOG^ mice is unspecifically expressed in cells other than B cells, and therefore leads to negative selection by non-B cells, we crossed B^MOG^/2D2 mice to B cell-deficient JHT mice. In JHT mice, the whole JH locus was deleted using the Cre/loxP system, leading to mice that are not capable to rearrange the heavy chain locus, and are therefore absolutely devoid of B cells [22]. As seen in Fig. 2, B^MOG^/2D2 mice crossed to the JHT mutant mice show a T cell development that is comparable to that of 2D2 WT mice. These findings demonstrate that the negative selection seen in B^MOG^/2D2 mice is indeed the result of the MOG peptide presented by MHCII of B cells, and not by unspecific expression of CD19-Cre, that would lead to Cre expression and thus MOG presentation by non-B cells in the thymus.

### T cells that escape negative selection express lower levels of the transgenic β chain

**Figure 3:**
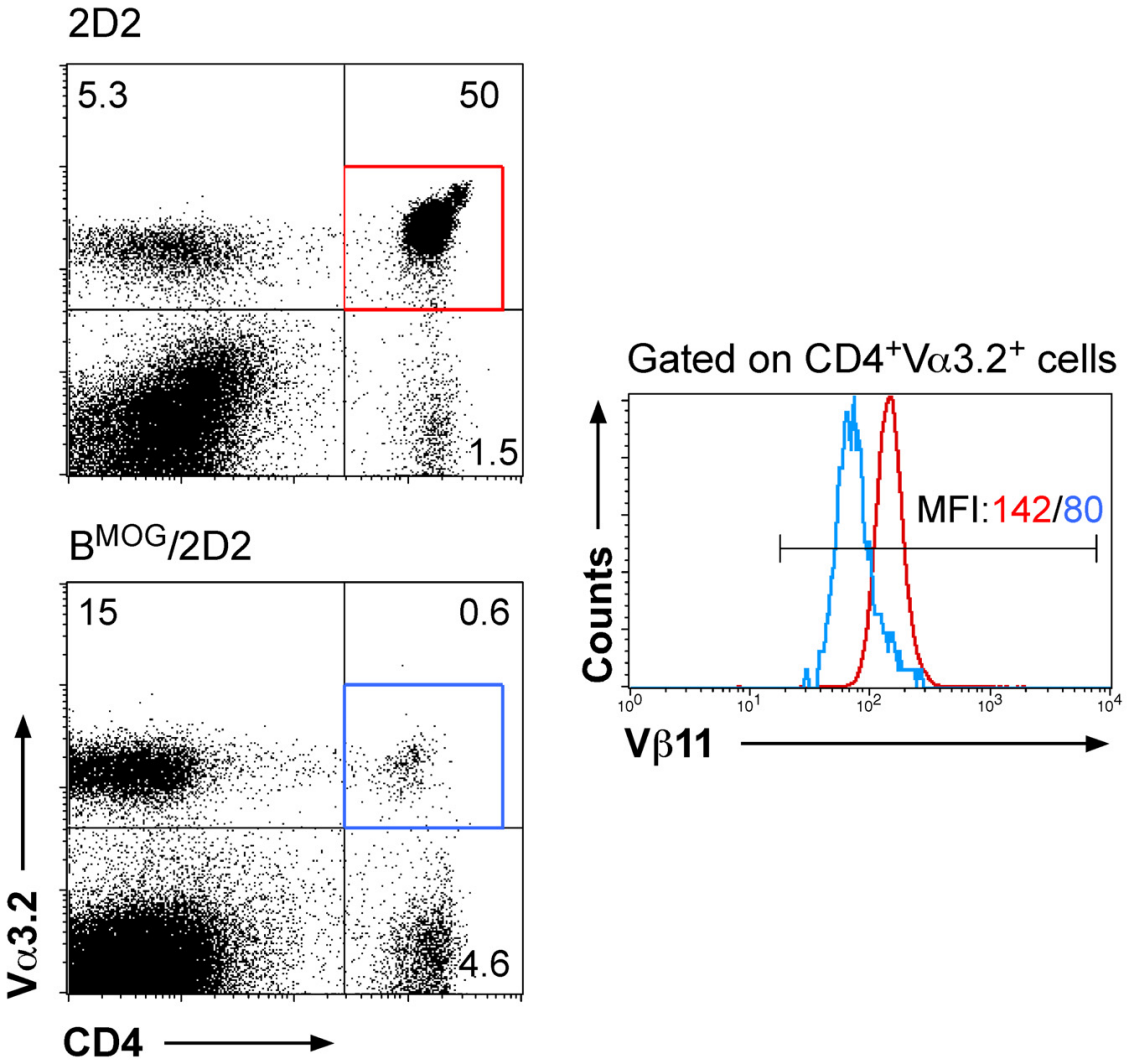
Residual MOG-specific T cells in B^MOG^/2D2 mice downregulate Vβ11. LN of WT 2D2 and B^MOG^/2D2 mice were analyzed by FACS analysis for presence of CD4, Vα3.2 and Vβ11. Cells were analyzed for expression of Vα3.2 vs CD4 (dot blots). Cells were then gated on CD4^+^ Vα3.2^+^ T cells and the percentage of Vβ11-expressing cells among this population is shown by histogram overlay. The red line in the histograms refers to the red gate of CD4^+^ Va3.2^+^ cells in 2D2 mice, the blue line refers to CD4^+^ Va3.2^+^ cells (blue gate) of B^MOG^/2D2 mice. Colored numbers above the marker line indicate MFI (mean fluorescence intensity) of anti-Vβ11-PE for the respective populations.

The number of CD4^+^Vα3.2^+^ T cells is dramatically reduced in the LN of B^MOG^/2D2 compared to 2D2 mice (Fig. 1 and 3). Interestingly, all surviving cells additionally still express the transgenic TCR β chain, namely Vβ11 (Fig. 3). But the expression levels of the TCR Vβ11 are reduced in mice where B cells present the MOG peptide (see histogram in Fig. 3). These findings suggest that the remaining transgenic TCR-expressing T cells can recognize the MOG peptide presented by B cells in the periphery, as reduced TCR expression is an indicative of recent T cell activation.

### Thymic regulatory T cells are spared from negative selection by B cells

**Figure 4:**
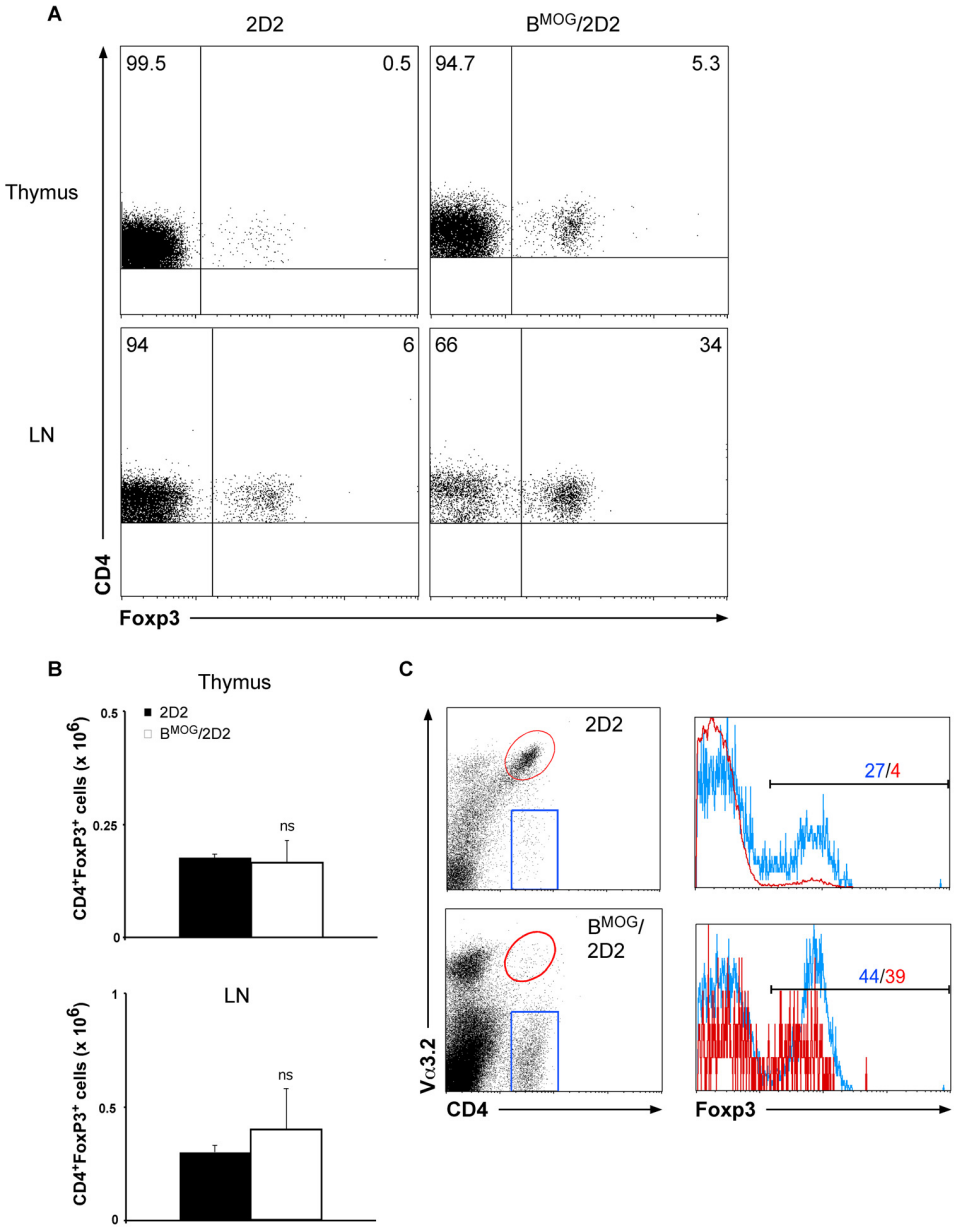
FACS analysis of Foxp3-expressing T cells in B^MOG^/2D2 mice. (A) Thymus and LN of WT 2D2 and B^MOG^/2D2 mice were analyzed for expression of Foxp3 by intracellular FACS staining. Genotypes and antibodies used are as indicated. Cells were gated on CD4^+^ cells. Numbers in quadrants indicate percent positive cells. (B) Total numbers of CD4^+^ Foxp3^+^ T cells from thymus and LN were calculated. Data represent mean values ± SEM of several individual experiments. ns, not significant. (C) LN cells from B^MOG^/2D2 and WT 2D2 mice were gated on CD4^+^ Va3.2^+^ or CD4+ Va3.2^−^ T cells, respectively, and percentage of Foxp3-expressing cells among these populations is shown by histograms. The blue line in the histograms refers to the blue gate of CD4^+^ Va3.2^−^ cells, the red line refers to CD4^+^ Va3.2^+^ cells (red gate). Colored numbers above the marker line indicate percent positive cells for the respective populations.

It was suggested that Foxp3 expression and thus development and function of regulatory T cells are dependent on the strength of TCR signaling during thymic selection [23]. To investigate whether the expression of self-peptide by B cells influences the number of regulatory T cells in the thymus, we analyzed the proportion of Foxp3^+^ T cells in B^MOG^/2D2 mice. As can be seen in Fig. 4A, the percentage of Foxp3^+^ T cells in the thymus of 2D2 mice is minimal, and accounts for only 0.5% of all CD4^+^ T cells. In contrast, we observed a 10-fold increase of Foxp3^+^ T cells in the thymus of B^MOG^/2D2 mice (Fig. 4A). Importantly, this increase is not due to increased total numbers of these cells (Fig. 4B), but reflects the decrease of the Foxp3^−^ T cell population. As can be seen in Fig. 4C (and Fig. 1–3), very few CD4^+^Vα3.2^+^ T cells can be found in the LN of B^MOG^/2D2 mice when compared to LN of 2D2 WT mice. The decrease of MOG-specific T cells in the peripheral lymphoid organs of B^MOG^/2D2 mice is most likely due to the decrease in MOG-specific CD4^+^ T cells in the thymus of these mice, and as mentioned above, is suggested to occur upon deletion of antigen-specific T cells that encounter B cells presenting the MOG peptide in the thymus. Interestingly, when we analyzed the percentage of Foxp3^+^ T cells among the Vα3.2-expressing and non-expressing T cells, we found that in single transgenic 2D2 mice, a large proportion of Vα3.2 negative cells express this essential regulatory T cell transcription factor (Fig. 4C). In fact, this proportion, 27%, is much higher than normally seen in WT mice. It is a strong indication that the process of TCR editing in the thymus is coupled to the process of regulatory T cell generation. This proportion of Foxp3-expressing T cells among the Vα3.2 negative cells is slightly increased in B^MOG^/2D2 mice (Fig. 4C). But most importantly, the proportion of Foxp3-expressing T cells among the Vα3.2-expressing T cells is also greatly enhanced (Fig. 4C), leading to mice in which about a third of all CD4^+^ T cells are Foxp3^+^.

### Peripheral T cells that have escaped negative selection by B cells are anergic

**Figure 5:**
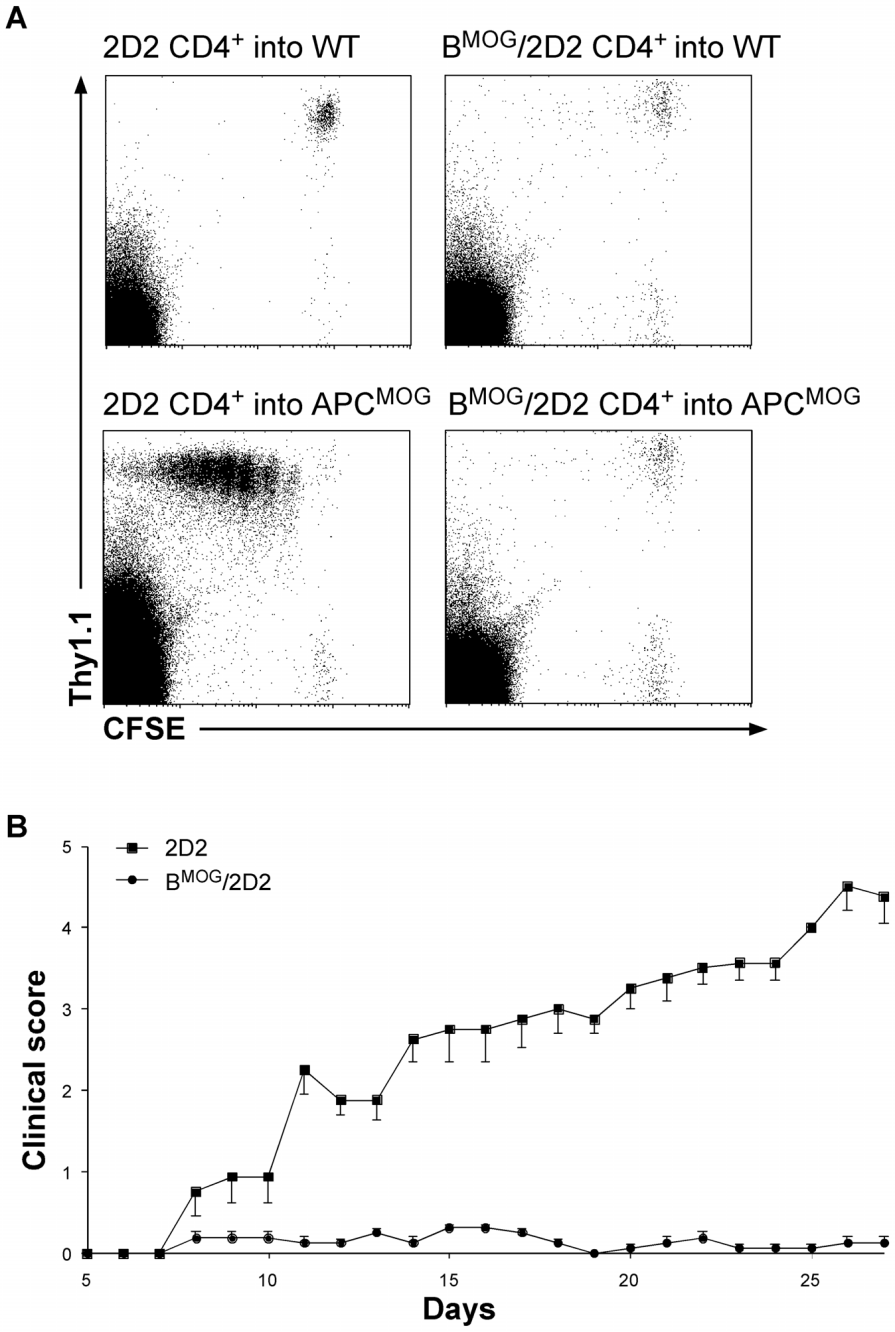
(**A**) **2D2 CD4^+^ T cells from B^MOG^/2D2 mice do not proliferate upon** ***in vivo*** **MOG stimulation.** CD4^+^ Thy1.1^+^ T cells from WT 2D2 and B^MOG^/2D2 mice were MACS-purified, CFSE-labeled, and transferred to APC^MOG^ mice (∼10×10^6^/mouse). Prior to transfer, APC^MOG^ mice were injected with anti-CD40. Five days after transfer, cells were monitored for proliferation by FACS analysis and loss of CFSE is depicted by representative dot blots of LN cells. Genotypes and antibodies used are as indicated. Cell surface markers are shown as coordinates. Cells were gated on live lymphocytes. (**B**)** B^MOG^/2D2 mice are resistant to EAE.** EAE was actively induced by immunization of 2D2 (squares) and B^MOG^/2D2 (circles) mice with MOGp35-55. B^MOG^/2D2 mice are protected from EAE compared to 2D2 WT mice (p<0,05: days 12–27). Values represent mean (± SEM) clinical scores. Shown is one representative of two individual experiments (n≥4 mice/group).

We have shown that B cells presenting the MOG peptide by MHCII can participate in the process of negative selection, leading to mice with very few T cells expressing the transgenic TCR. Next, we investigated whether the relatively few remaining T cells are capable of recognizing the MOG peptide *in vivo*, or whether they are anergic. To this end, we have isolated T cells from LN and spleen of B^MOG^/2D2 mice and transferred them into mice where the MOG peptide is presented by all APCs (APC^MOG^ [19]). As a control, we transferred CD4^+^ T cells isolated from 2D2 mice. As can be seen in Fig. 5A, T cells of 2D2 mice proliferated extensively once transferred to mice where the MOG peptide is expressed by all APCs, as we have demonstrated previously [19]. In contrast, we could not detect proliferation of T cells from B^MOG^/2D2 mice, and they behaved identically when transferred to mice where the MOG peptide is not expressed (Fig. 5A). Finally, we have attempted to induce EAE in B^MOG^/2D2 mice. Normally, 2D2 mice are highly susceptible to the MOG-induced disease, as the vast majority of the naïve T cell repertoire in these mice is MOG-specific [20]. Indeed, we noted that the 2D2 mice all developed EAE, once immunized with the MOG peptide (Fig. 5B). In contrast, B^MOG^/2D2 mice were completely resistant to EAE induction (Fig. 5B). These data indicate that the relatively low number of CD4^+^ T cells that remain in B^MOG^/2D2 mice are either tolerized or their number is just not sufficient for disease induction and as a result, not able to induce the development of EAE.

## Discussion

Elimination of self-reactive T cells in the thymus is a critical mechanism of immunological tolerance. B cells can be found in the thymus, but their participation in negative selection of self-reactive thymocytes has been poorly addressed. In his study, we show that B cells that are enforced to present an autoantigen by their MHCII molecules induce strong deletion of self-reactive T cells and that this process results in complete resistance to EAE. Further, we demonstrate that the process of negative selection by B cells is solely effective for conventional CD4^+^ T cells, but spares regulatory CD4^+^FoxP3^+^ T cells, thus resulting in an imbalance between effector and regulatory T cells.

Promiscuous gene expression by mTECs was shown to be crucial for thymic negative selection of self-reactive T cells [6], [7], [8], but also thymic DCs have an important role in negative selection [14], [18], [24]. In contrast, conventional B-2 as well as B-1 B cells have previously been reported to reside in the thymus [21], but their exact function was not clearly shown. We have demonstrated that B cells that are engineered to express the MOG peptide p35-55 on MHCII interact with MOG-specific CD4^+^ 2D2 T cells in a process that eventually results in the deletion of naïve T cells, as in the periphery of these mice only few MOG-specific T cells can be found. However, a role of B cells in thymic negative selection was so far only clearly evidenced by [18]. In this study, they targeted expression of I-Eα self-antigen to the B cell lineage using the human CD19 promoter and studied negative selection in the I-E negative C57Bl/6 background. Depending on the TCR β chain, they observed deletion of CD4^+^ T cells of 36 up to 42% [18].

Our study not only shows a partial deletion of self-reactive T cells but the nearly entire elimination of MOG-specific T cells in B^MOG^ mice. Indeed, the deletion accounts for over 95% of T cells in B^MOG^/2D2 mice when compared to 2D2 controls. This strong effect might display the potential importance of B cells in the process of negative selection or might in part also be due to the high proportion of B cells presenting the peptide on MHCII. However, the number of B cells in the thymus is generally not high (less than 1% of total lymphocytes; [21]). Thus, we speculate that the strong deletion of 2D2 T cells in B^MOG^/2D2 mice results from the B cells' potential of negatively selecting self-reactive thymocytes.

Although CD19-Cre mice were generated over 20 years ago [25] and successfully used in numerous studies since then [26], [27], [28], [29], [19] the use of Cre-expressing mouse strains carries along the risk of unspecific Cre activity in cell types other than the requested ones and thus might lead to misleading results [30]. To exclude this possibility, we crossed B^MOG^/2D2 mice on a B cell-deficient background using JHT mice [22] and compared CD4^+^ T cell development in the thymus of these mice to all controls. We could demonstrate normal CD4^+^ T cell development (comparable to 2D2 WT mice) and no negative selection of MOG-specific T cells in B^MOG^/2D2/JHT mice and thus concluded that negative selection in B^MOG^/2D2 mice is indeed due to Cre expression in B cells of these mice and not due to unspecific Cre activity in other cell types of the thymus.

As seen in Fig. 1, 3 and 4, we noted a population of Vα3.2^+^CD4^−^ T cells in LN of both, 2D2 and B^MOG^/2D2 mice. The percentage, but not the total cell number of this population is increased in B^MOG^/2D2 mice compared to 2D2 WT mice and generally varies between independent experiments. Moreover, these cells are CD8^−^ (data not shown). This phenomenon was seen for different TCR transgenic mice, especially for those carrying an autoreactive TCR and these cells were shown to be TCRαβ^+^CD4^−^ and either CD8^low^ or CD8^−^ [31]. It was considered that these cells are γδ T cells that never accomplished γδ TCR rearrangement, but are instead positive for the transgenic αβ TCR chain (‘γδ wannabe T cells’, [31]). Since such a population was found in HY TCR transgenic males (where HY-specific T cells are negatively selected) it was speculated that these cells might be escapees from negative selection [31]. Accordingly, the population of Vα3.2^+^CD4^−^CD8^−^ cells, which we observe notably in B^MOG^/2D2 mice, might be T cells that have escaped negative selection by B cells.

Importantly, we also analyzed B^MOG^/2D2 mice for the development of EAE after standard immunization with MOGp35-55 and observed all of these mice being completely resistant to disease. The resistance is probably due to the fact, that over 95% of the MOG-specific T cell are deleted in the thymi of B^MOG^/2D2 mice, thus leaving behind T cell numbers that are not sufficient for the development of EAE. However, some T cells escape negative selection by B cells. To investigate whether these cells are still functional, we subjected them to stimulation with MOG by all APCs (transfer to APC^MOG^ mice). The remaining MOG-specific T cells were not able to proliferate when stimulated *in vivo* by MOG-presenting APCs. This is in line with our previously published results where we showed that B cells induce peripheral tolerance of auto-reactive T cells [19].

Our study further extends to the analysis of regulatory T cells (Tregs) and we observed that the population of CD4^+^Foxp3^+^ Tregs remains apparently unaffected in our experimental mice. Consequently, we noted an increased proportion but unchanged total cell numbers of Tregs since the auto-reactive MOG-specific T cells were deleted but not the Tregs. Due to the fact that the total cell numbers are unaltered, we conclude that Tregs are more resistant to apoptosis and thus accumulate when self-reactive conventional T cells are negatively selected. Whether or not Tregs are more apoptosis-resistant was under debate in the last years and some studies reported a normal sensitivity of Tregs to thymic deletion [32], [33]. However, in line with our data, it was shown that CD4^+^CD25^+^ thymocytes are much less sensitive to agonist-induced clonal deletion than CD4^+^CD25^−^ thymocytes [34]. This explains the accumulation of Tregs in the presence of agonist ligand being due to their enrichment by relative resistance to apoptosis, rather than to induced differentiation. Also, a study by Taylor et al. showed self-reactive Tregs being moderately resistant to TCR-mediated apoptosis, resulting in a bias of Treg TCRs towards autoreactivity in comparison to TCRs of conventional T cells [35]. Our data suggests that the elevated Treg proportions in B^MOG^/2D2 mice account for their relative resistance to apoptosis while conventional T cells are deleted due to the MOG presentation by B cells.

## Materials and Methods

### Mice

All animal experiments were in accordance with the guidelines of the central animal facility institution (ZVTE, University of Mainz), and approved accordingly to animal protection law with permissions number AZ 177-07/051-2. 2D2, CD19-Cre and IiMOG mice were described elsewhere [20], [25], [19]. DNA for typing was prepared from tail biopsies. Presence of the different alleles was routinely tested by PCR as published for CD19-Cre [25], [36], 2D2 [20] and IiMOG mice [19]. All experiments were performed with 6–12 weeks old mice. The experiments were repeated at least once.

### Cell preparations and CFSE staining

2D2 CD4^+^ T cells were isolated from spleen and LN by positive selection using MACS (Miltenyi Biotech) according to the manufacturer's instructions. Purity of the resulting CD4^+^ T cells was typically >95%. When indicated freshly isolated CD4^+^ T cells from 2D2 mice were labeled with the vital dye CFSE (Molecular Probes). After washing the cells twice in 10 ml PBS, pH 7.4, they were incubated with 0.5 mM CFSE in 1 ml PBS per 10^7^ cells at RT for 8 min. To stop the staining reaction 8 ml RPMI plus 10% FCS was added. The cells were then washed twice in 10 ml RPMI, resuspended in the appropriate volume of PBS and typically 5–10×10^6^ T cells were injected i.v. into the different mice as indicated. When indicated recipient mice received an i.p. injection of 50 µg agonistic anti-CD40 (FGK45.5, [37], homemade).

### Flow cytometry

For cytofluorometric analysis we used Ab conjugates to the following antigens: CD4, CD8, Thy1.1, Vα3.2, Vβ11. Antibodies were obtained from BD Pharmingen or Natutec (eBiosciences). Intracellular stainings for Foxp3 were performed using the Foxp3 Staining Set (Natutec, eBiosciences) according to the manufacturer's instructions. Cells from lymphoid organs were stained with the Ab conjugates for flow cytometric analysis on a FACSCalibur or a FACScan (BD Biosciences). Events in a live lymphocyte gate were analyzed with CellQuest (BD Biosciences) software.

### Induction and assessment of EAE

MOG_35–55_ peptide (amino acid sequence: MEVGWYRSPFSRVVHLYRNGK) was obtained from Research Genetics. Active EAE was induced by immunization with 50 µg of MOG_35–55_ peptide emulsified in CFA (Difco Laboratories) supplemented with 10 mg/ml of heat-inactivated *Mycobacterium tuberculosis* H37RA (Difco Laboratories). The emulsion was administered as a 100 µl subcutaneous injection in the tail base. Mice also received 200 ng of pertussis toxin (Sigma Aldrich) intraperitoneally (i.p.) on the day of immunization and 2 days later. Clinical assessment of EAE was performed daily according to the following criteria: 0, no disease; 1, decreased tail tone; 2, abnormal gait (ataxia) and/or impaired righting reflex (hind limb weakness or partial paralysis); 3, partial hind limb paralysis; 4, complete hind limb paralysis; 5, hind limb paralysis with partial fore limp paralysis; 6, moribund or dead.

### Statistics

Values are presented as mean ± SEM. Statistical significance was assessed using 2-tailed Student's *t*-test. p-values <0.05 were regarded significant.

## References

[1] Anderson MS, Venanzi ES, Klein L, Chen Z, Berzins SP (2002). Projection of an immunological self shadow within the thymus by the aire protein.. Science.

[2] Ramsey C, Winqvist O, Puhakka L, Halonen M, Moro A (2002). Aire deficient mice develop multiple features of APECED phenotype and show altered immune response.. Hum Mol Genet.

[3] Liston A, Lesage S, Wilson J, Peltonen L, Goodnow CC (2003). Aire regulates negative selection of organ-specific T cells.. Nat Immunol.

[4] Nagamine K, Peterson P, Scott HS, Kudoh J, Minoshima S (1997). Positional cloning of the APECED gene.. Nat Genet.

[5] Klein L, Klugmann M, Nave KA, Tuohy VK, Kyewski B (2000). Shaping of the autoreactive T-cell repertoire by a splice variant of self protein expressed in thymic epithelial cells.. Nat Med.

[6] Doffinger R, Klein TC, Pepys MB, Casanova JL, Kyewski BA (1997). The MHC class II-restricted T cell response of C57BL/6 mice to human C-reactive protein: homology to self and the selection of T cell epitopes and T cell receptors.. Mol Immunol.

[7] Derbinski J, Schulte A, Kyewski B, Klein L (2001). Promiscuous gene expression in medullary thymic epithelial cells mirrors the peripheral self.. Nat Immunol.

[8] Hanahan D (1998). Peripheral-antigen-expressing cells in thymic medulla: factors in self-tolerance and autoimmunity.. Curr Opin Immunol.

[9] Volkmann A, Zal T, Stockinger B (1997). Antigen-presenting cells in the thymus that can negatively select MHC class II-restricted T cells recognizing a circulating self antigen.. J Immunol.

[10] Surh CD, Sprent J (1994). T-cell apoptosis detected in situ during positive and negative selection in the thymus.. Nature.

[11] Fadok VA, Voelker DR, Campbell PA, Cohen JJ, Bratton DL (1992). Exposure of phosphatidylserine on the surface of apoptotic lymphocytes triggers specific recognition and removal by macrophages.. J Immunol.

[12] Hare KJ, Pongracz J, Jenkinson EJ, Anderson G (2003). Modeling TCR signaling complex formation in positive selection.. J Immunol.

[13] Jenkinson EJ, Anderson G, Moore NC, Smith CA, Owen JJ (1994). Positive selection by purified MHC class II+ thymic epithelial cells in vitro: costimulatory signals mediated by B7 are not involved.. Dev Immunol.

[14] Gallegos AM, Bevan MJ (2004). Central tolerance to tissue-specific antigens mediated by direct and indirect antigen presentation.. J Exp Med.

[15] van Meerwijk JP, Marguerat S, Lees RK, Germain RN, Fowlkes BJ (1997). Quantitative impact of thymic clonal deletion on the T cell repertoire.. J Exp Med.

[16] Proietto AI, van Dommelen S, Zhou P, Rizzitelli A, D'Amico A (2008). Dendritic cells in the thymus contribute to T-regulatory cell induction.. Proc Natl Acad Sci U S A.

[17] Ohnmacht C, Pullner A, King SB, Drexler I, Meier S (2009). Constitutive ablation of dendritic cells breaks self-tolerance of CD4 T cells and results in spontaneous fatal autoimmunity.. J Exp Med.

[18] Kleindienst P, Chretien I, Winkler T, Brocker T (2000). Functional comparison of thymic B cells and dendritic cells in vivo.. Blood.

[19] Frommer F, Heinen TJ, Wunderlich FT, Yogev N, Buch T (2008). Tolerance without clonal expansion: self-antigen-expressing B cells program self-reactive T cells for future deletion.. J Immunol.

[20] Bettelli E, Pagany M, Weiner HL, Linington C, Sobel RA (2003). Myelin oligodendrocyte glycoprotein-specific T cell receptor transgenic mice develop spontaneous autoimmune optic neuritis.. J Exp Med.

[21] Akashi K, Richie LI, Miyamoto T, Carr WH, Weissman IL (2000). B lymphopoiesis in the thymus.. J Immunol.

[22] Gu H, Zou YR, Rajewsky K (1993). Independent control of immunoglobulin switch recombination at individual switch regions evidenced through Cre-loxP-mediated gene targeting.. Cell.

[23] Fontenot JD, Gavin MA, Rudensky AY (2003). Foxp3 programs the development and function of CD4+CD25+ regulatory T cells.. Nat Immunol.

[24] Brocker T, Riedinger M, Karjalainen K (1997). Targeted expression of major histocompatibility complex (MHC) class II molecules demonstrates that dendritic cells can induce negative but not positive selection of thymocytes in vivo.. J Exp Med.

[25] Rickert RC, Roes J, Rajewsky K (1997). B lymphocyte-specific, Cre-mediated mutagenesis in mice.. Nucleic Acids Res.

[26] Anzelon AN, Wu H, Rickert RC (2003). Pten inactivation alters peripheral B lymphocyte fate and reconstitutes CD19 function.. Nat Immunol.

[27] Li ZW, Omori SA, Labuda T, Karin M, Rickert RC (2003). IKK beta is required for peripheral B cell survival and proliferation.. J Immunol.

[28] Sasaki Y, Derudder E, Hobeika E, Pelanda R, Reth M (2006). Canonical NF-kappaB activity, dispensable for B cell development, replaces BAFF-receptor signals and promotes B cell proliferation upon activation.. Immunity.

[29] Hovelmeyer N, Wunderlich FT, Massoumi R, Jakobsen CG, Song J (2007). Regulation of B cell homeostasis and activation by the tumor suppressor gene CYLD.. J Exp Med.

[30] Schmidt-Supprian M, Rajewsky K (2007). Vagaries of conditional gene targeting.. Nat Immunol.

[31] Bruno L, Fehling HJ, von Boehmer H (1996). The alpha beta T cell receptor can replace the gamma delta receptor in the development of gamma delta lineage cells.. Immunity.

[32] Hsieh CS, Zheng Y, Liang Y, Fontenot JD, Rudensky AY (2006). An intersection between the self-reactive regulatory and nonregulatory T cell receptor repertoires.. Nat Immunol.

[33] Romagnoli P, Hudrisier D, van Meerwijk JP (2002). Preferential recognition of self antigens despite normal thymic deletion of CD4(+)CD25(+) regulatory T cells.. J Immunol.

[34] van Santen HM, Benoist C, Mathis D (2004). Number of T reg cells that differentiate does not increase upon encounter of agonist ligand on thymic epithelial cells.. J Exp Med.

[35] Taylor SR, Alexander DR, Cooper JC, Higgins CF, Elliott JI (2007). Regulatory T Cells Are Resistant to Apoptosis via TCR but Not P2X7.. J Immunol.

[36] Rickert RC, Rajewsky K, Roes J (1995). Impairment of T-cell-dependent B-cell responses and B-1 cell development in CD19-deficient mice.. Nature.

[37] Rolink A, Melchers F, Andersson J (1996). The SCID but not the RAG-2 gene product is required for S mu-S epsilon heavy chain class switching.. Immunity.

